# In-center Nocturnal Hemodialysis Reduced the Circulating FGF23, Left Ventricular Hypertrophy, and All-Cause Mortality: A Retrospective Cohort Study

**DOI:** 10.3389/fmed.2022.912764

**Published:** 2022-06-21

**Authors:** Meizi Kang, Jing Chen, Lingling Liu, Cheng Xue, Xiaojing Tang, Jiayi Lv, Lili Fu, Changlin Mei, Zhiguo Mao, Yawei Liu, Bing Dai

**Affiliations:** ^1^Division of Nephrology, The Second Affiliated Hospital of Nantong University, Nantong, China; ^2^Division of Nephrology, Kidney Institute of People's Liberation Army of China, Shanghai Changzheng Hospital, Naval Medical University, Shanghai, China; ^3^Department of Internal Medicine, Shanghai Changzheng Hospital, Naval Medical University, Shanghai, China

**Keywords:** in-center nocturnal hemodialysis, fibroblast growth factor 23, Chronic Kidney DiseaseMineral and Bone Disorder (CKD-MBD), calcium-phosphate product, left ventricular hypertrophy

## Abstract

Fibroblast growth factor 23(FGF23) is the most important biomarker and pathogenic factor in Chronic Kidney Disease–Mineral and Bone Disorder (CKD–MBD). In the moderate and severe stages of chronic renal failure, abnormally elevated circulating FGF23 can lead to some complications, including myocardial hypertrophy, which is positively correlated with all-cause mortality. However, the circulating FGF23 level of different hemodialysis modalities, the underlying essential regulatory factors, and potential clinical benefits remain to be elucidated. In this retrospective cohort study, 90 in-center nocturnal hemodialysis (INHD) and 90 matched conventional hemodialysis (CHD) patients were enrolled. The complete blood count, intact FGF23(iFGF23), calcium, phosphorus, PTH, and other biochemical and echocardiographic parameters of INHD and CHD patients were collected and analyzed at 1-year follow-up. The all-cause mortality was recorded during the 7-year follow-up. Furthermore, the regulatory factors of iFGF23 and its association with echocardiographic parameters and mortality were investigated by multivariate regression. The levels of iFGF23 and serum phosphate in patients undergoing INHD were significantly lower than those in patients undergoing CHD. The left ventricular volume index (LVMI) in patients with INHD was significantly attenuated and positively correlated with the drop of serum iFGF23. The INHD group had reduced all-cause mortality compared to the CHD group. Multivariate analysis showed that iFGF23 was positively correlated with serum calcium, serum phosphorus, and calcium-phosphate product. The calcium-phosphate product is an independent determining factor of serum iFGF23. Compared with the CHD group, the INHD group presented with a significantly reduced circulating iFGF23 level, which was closely associated with attenuation of left ventricular hypertrophy, but INHD reduced all-cause mortality in an FGF23 independent manner.

## Introduction

Fibroblast growth factor 23 (FGF23) is the most important biomarker and pathogenic factor in CKD–MBD, which is one of the most common complications in dialysis patients. As a potent calcium and phosphorus regulator, FGF23 is produced by osteoblasts and osteocytes. It can reduce phosphorus by promoting phosphorus excretion and inhibiting the formation of 1,25(OH)_2_D_3_ (1). In the early stage of chronic renal failure, FGF23 secretion is stimulated by an uncertain mechanism to maintain phosphorus balance, subsequently, leading to decreased 1,25(OH)_2_D_3_ synthesis and triggering secondary hyperparathyroidism (2). With the deterioration of renal function, its capacity to promote phosphorus excretion gradually lessens due to reduced functional nephrons, abnormally elevated FGF23 instead becomes a uremic toxin and is highly associated with adverse clinical outcomes (3). So, FGF23 has been recognized as an important prognosis biomarker of chronic kidney disease (4–6).

Hemodialysis is the most widely used modality for renal replacement therapy, which has greatly improved the prognosis of patients with uremia (7). In contrast to conventional hemodialysis, which is performed three times a week for 4 h each, intensive dialysis includes daily dialysis, in-center nocturnal hemodialysis, and home nocturnal hemodialysis (8). The mortality of conventional hemodialysis (CHD) patients is still relatively high, it is ~4 times more than healthy people under the age of 30, and six times more than healthy people over the age of 65 (9). Intensive dialysis has been reported to have many clinical benefits, including improved blood pressure, anemia, serum phosphorus, and reduced left ventricular hypertrophy (10). A large multicenter study showed that patients with INHD have a higher quality of life, prolonged survival, and a lower hospital admission rate (11). In our previous studies, regression of left ventricular mass index (LVMI) also had been detected when patients with ESRD convert from CHD to INHD (12, 13).

Since elevated FGF23 was found to be associated with increased cardiovascular mortality and all-cause mortality (4), we speculated that INHD might attenuate left ventricular hypertrophy and reduce mortality through lowering the serum FGF23 due to modification of CKD–MBD parameters. However, the effect of different hemodialysis modalities on the circulating FGF23 levels, the underlying essential regulatory mechanism, and potential clinical benefits remain to be elucidated. This study is aimed to further explore whether INHD can improve the prognosis by reducing the level of FGF23. We performed this retrospective cohort study to compare serum FGF23, CKD–MBD related parameters, hemoglobin, albumin, echocardiographic, and survival data in patients undergoing CHD and INHD. The regulatory factors of FGF23 and their association with echocardiographic parameters and mortality were investigated by multivariate regression.

## Patients and Methods

### Study Design and Population

This retrospective study included prevalent long-term hemodialysis patients aged over 18 years old at Shanghai Changzheng Hospital in January 2015. Patients diagnosed with malignant tumors, serious infections or severe heart failure, and liver failure were excluded. A total of 90 patients were enrolled and converted from CHD to INHD. In total, 90 matched patients were enrolled and persistently received CHD (Supplementary Figure S1). They were followed up until February 2022. This study was approved by the Ethics Committee of Changzheng Hospital. All patients were dialyzed on a Rexeed 15uc dialyzer (polysulfone membrane, 1.5 m^2^). INHD patients received thrice-weekly hemodialysis for 7.5 h each session. Blood flow rates were 200–300 ml/min. Dialysate flow rates were 300–500 ml/min. CHD patients received thrice-weekly hemodialysis (or twice hemodialysis plus single hemodiafiltration) for 4 h each session. Blood flow rates were 200–360 ml/min. Dialysate flow rates were 500 ml/min. Anticoagulation was achieved with heparin or low-molecular-weight heparin.

### Data Collection

The demographic data, dialysis vintage, and primary diseases of ESRD were collected from the electronic database of our hemodialysis center. The baseline biochemical parameters, including calcium (Ca), phosphorus (P), hemoglobin (Hb), albumin (Alb), serum creatinine (Scr), ferritin, lipid profiles, 25-hydroxyvitamin D, intact parathyroid hormone (iPTH), and KT/V were collected. The blood samples for laboratory tests, including iFGF23, were obtained before the midweek hemodialysis session. Serum intact FGF23 concentrations with biological activity were measured using a human FGF23 ELISA assay kit (Kainos Lab, Tokyo, Japan). Medications during the enrollment period, including antihypertensive drugs, erythropoietin, phosphate binders, calcitriol, and intravenous iron were reviewed. One year later after enrollment, all the laboratory tests and medications were evaluated again. M-mode echocardiography was used to measure the echocardiographic parameters of 40 matched patients in each group, including interventricular septum thickness diastolic (IVSTd), left ventricular end-diastolic diameter (LVDd), and left ventricular posterior wall thickness (LVPWT), etc. The echocardiography data together with total ultrafiltration volume (ml), the ultrafiltration rate (ml/h/kg), and BP were collected from the electronic database at 1-year follow-up. LVMI was calculated according to the Devereux formula. Left ventricular mass (LVM) = 1.04 × [(IVSTd + LVDd + LVPWT)^3^ – LVDd^3^] −13.6, body surface area (BSA) = 0.0061 × height (cm) + 0.0128 × weight (kg) – 0.1529. LVMI = LVM/BSA. The all-cause mortality and survival data of the two groups were reviewed during 7 year follow-up.

### Statistical Analysis

A propensity-score matching (PSM) analysis was performed to adjust for patient selection. All the quantitative data were presented as mean ± SD. The non-normally distributed variable iFGF-23 was described as medians (interquartile range) and ln-transformed to achieve a normal distribution. The laboratory test data of the two groups were analyzed by the independent sample *t*-test. The association of iFGF23 with patient demographics and laboratory variables was evaluated by Pearson correlation analysis. The correlations between iFGF23 and potential regulatory factors were analyzed by multivariate regression, which was typically shown by some scatter plots. The association of left ventricular volume index (LVMI) with iFGF23 in two groups and overall patients was detected by univariate correlation analysis. We adopted the Kaplan–Meier analysis (log-rank method) to evaluate the survival differences between the two groups. A multivariable Cox proportional hazards regression model was used to identify the risk factors for all-cause mortality. To better clarify the association between iFGF23 and mortality, adjusted HR (95% CI) for mortality was analyzed by both lnFGF23 and quartiles of FGF23 using the first quartile as the reference.

## Results

### Characteristics of Patients

This retrospective cohort study included 90 INHD patients with an average age of 53.23 ± 10.26 and 90 CHD patients with an average age of 53.32 ± 9.53. The baseline characteristics of the study subjects are shown in Table 1. After PSM, there was no significant difference in age, gender, dialysis vintage, primary disease, medications, and biochemical parameters, including serum calcium, phosphorus PTH, and iFGF23 (Supplementary Table S1) between the two groups.

**Table 1:** Baseline characteristics of patients undergoing hemodialysis after PSM.

**Item**	**INHD (*n* = 90)**	**CHD (*n* = 90)**
Sex		
Male	42	43
Female	48	47
Age (year)	53.23 ± 10.26	53.32 ± 9.53
Dialysis vintages (year)	8.32 ± 5.25	8.43 ± 5.12
Primary disease		
CGN	56	57
Hypertensive renal sclerosis	12	13
DN	15	14
Obstructive nephropathy	1	2
PKD	4	3
Others	2	1

*CGN, chronic glomerulonephritis; DN, diabetic nephropathy; PKD, polycystic kidney disease l*.

### Biochemical Parameters and Medications at 1-Year Follow-Up

The serum iFGF23 level of patients in the INHD group was significantly lower than that in the CHD group, as presented by lnFGF23 (7.57 ± 1.62 vs. 8.56 ± 1.17) (*P* < 0.05). INHD patients had lower mean phosphorus levels (1.42 ± 0.41 vs. 2.03 ± 0.62 mmol/L) and calcium-phosphate product levels (3.47 ± 1.02 vs. 5.0 ± 1.52 mmol^2^/L^2^). The good control rate within the KDIGO recommendation target of serum calcium in the INHD group was higher than that in the CHD group (61.2 vs. 59%). The INHD group exhibited a higher good control rate of phosphorus (69.2 vs. 52.9%) and iPTH (73.3 vs. 63%). There was no significant difference in calcium, albumin, 25-(OH)D, and hemoglobin levels between the two groups. Patients undergoing INHD had a higher mean KT/V than those undergoing CHD (2.30 ± 0.71 vs. 1.53 ± 0.30), representing better dialysis adequacy. Compared with CHD, patients in the INHD group used fewer types of antihypertensive drugs on average, and the dosage of erythropoietin and phosphate binder was lower. There was no difference in the dosage and number of patients who used calcitriol and intravenous iron as shown in Table 2.

**Table 2:** Comparison of parameters between CHD and INHD at 1-year follow-up.

**Parameters**	**INHD (*n* = 90)**	**CHD (*n* = 90)**
iFGF23 (pg·ml^−1^)	3,114.41 (1,023.23–9,582.05)	9,626.18 (4,258.53–10,123.23)
LnFGF23	7.57 ± 1.62^*^	8.56 ± 1.17
Pitch	306.02 ± 142.08	412.92 ± 212.10
Cholesterol (mmol·L^−1^)	3.82 ± 0.97	3.73 ± 0.92
Triglycerides (mmol·L^−1^)	2.25 ± 1.52	2.24 ± 1.45
LDL cholesterol (mmol·L^−1^)	2.08 ± 0.55	2.04 ± 0.60
HDL cholesterol (mmol·L^−1^)	0.89 ± 0.29	0.87 ± 0.27
Phosphate (mmol·L^−1^)	1.42 ± 0.41^*^	2.03 ± 0.62
Calcium (mmol·L^−1^)	2.44 ± 0.22	2.47 ± 0.33
Calcium-phosphate product (mmol^2^·L^−2^)	3.47 ± 1.02^*^	5.00 ± 1.52
Albumin (g·L^−1^)	42.32 ± 5.37	42.21 ± 4.38
KT/V	2.30 ± 0.71^*^	1.53 ± 0.30
25(OH)D (ng·ml^−1^)	27.6 ± 9.3	27.6 ± 9.3
Hemoglobin (g·L^−1^)	117.98 ± 13.76	111.69 ± 14.09
Ferritin (ug·L^−1^)	195.53 ± 159.28	237.30 ± 173.21
β2-MG (mg·L^−1^)	16.7 ± 3.3	17.4 ± 4.7
**Medications**		
Kinds of antihypertensive drugs/pts number	1.1 ± 0.3^*^/68	1.8 ± 0.5/74
EPO (U/week)/pts number	5,280.0 ± 3,120.0^*^/63	6,841.0 ± 3,210.0/70
CaCO_3_(g/d)/pts number	1.6 ± 1.2^*^/51	2.5 ± 1.0/53
Calcitriol (ug/d)/pts number	0.23 ± 0.19/49	0.22 ± 0.14/52
Iron sucrose (mg)/pts number	950 ± 296/16	964 ± 285/18
Iron dextran (mg)/pts number	783 ± 182/5	800 ± 167/6

*IFGF23, intact Fibroblast growth factor 23; IPTH, intact parathyroid hormone; LDL, low-density lipoprotein; HDL, high-density lipoprotein; KT/V, urea clear index; 25-(OH)D, 25-Hydroxy vitamin D; β2-MG, β2-Microglobulin.*

* *P <0.05*.

### Regulatory Factors of FGF23

**Figure 1:**
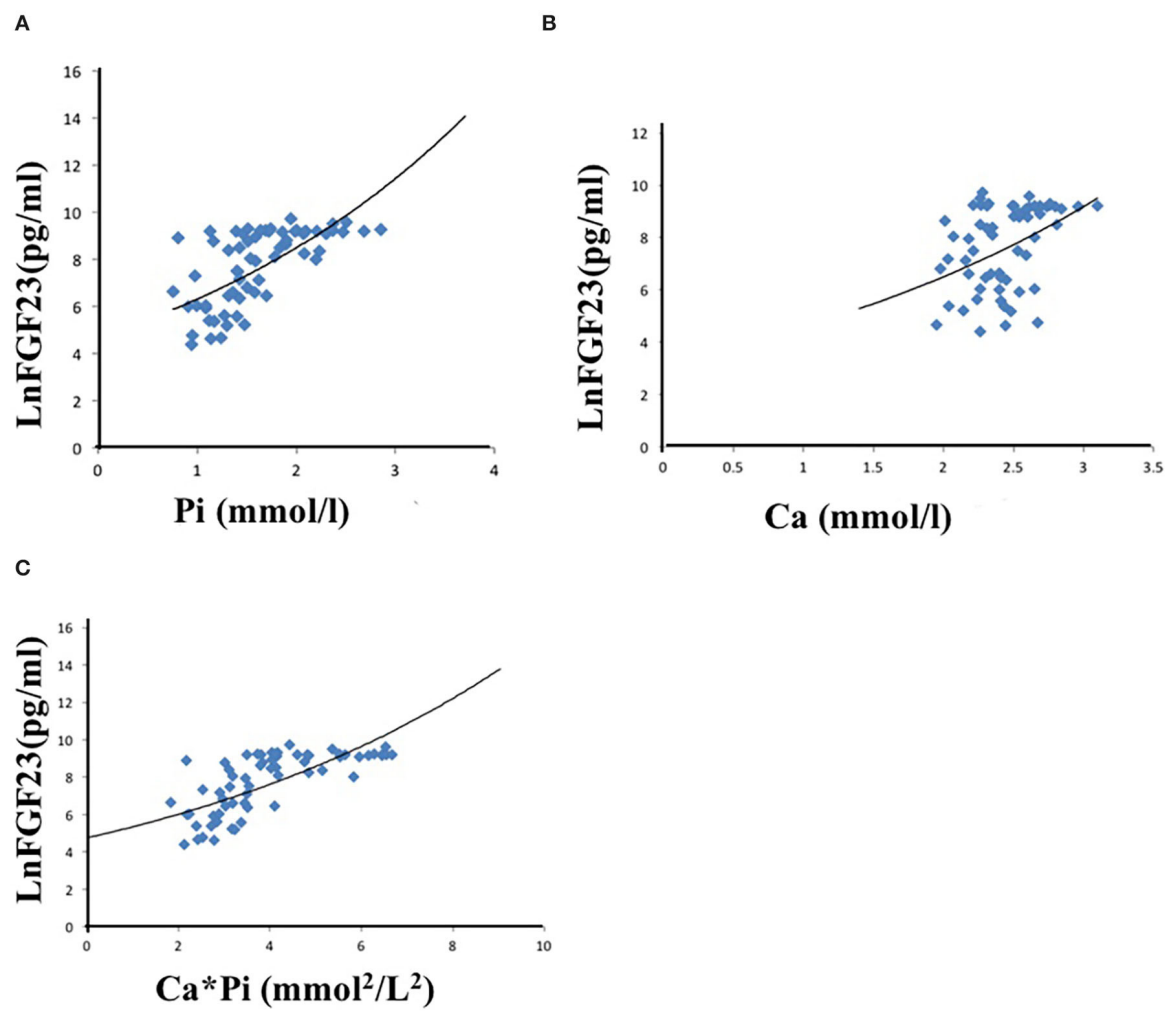
Univariate linear correlation between phosphate **(A)**, calcium **(B)**, calcium-phosphate product **(C)**, and FGF23.

Univariate linear regression analysis showed that serum phosphate (*r* = 0.480, *p* < 0.001), calcium (*r* = 0.413, *P* < 0.001), calcium-phosphate product (*r* = 0.593, *P* < 0.001), KT/V (*r* = −0.395, *P* < 0.001), and iPTH (*r* = 0.334, *P* < 0.001) were significantly associated with iFGF23 levels (Table 3). Among all the parameters, multivariable regression analysis showed that serum phosphate (β = 0.429, *P* < 0.001), calcium (β = 0.354, *P* = 0.001), calcium-phosphate product (β = 0.600, *P* < 0.001), but not KT/V (β = −0.218, *P* = 0.053) and iPTH (β = 0.235, *P* = 0.353) independently correlated with iFGF23 levels (Table 4). The scatter plots showed that lnFGF23 was linear with serum calcium, phosphorus, and calcium-phosphate product (Figure 1).

**Table 3:** Univariate correlation analysis of FGF23 and other parameters.

**Variables**	***r***	***T***	***P***
Age	0.432	0.702	0.484
Dialysis vintage	−0.182	1.823	0.071
Triglycerides	0.211	2.090	0.093
Cholesterol	0.186	1.839	0.069
LDL cholesterol	0.196	1.864	0.066
HDL cholesterol	−0.091	0.856	0.394
Phosphate	0.480	5.388	<0.001^*^
Calcium	0.413	4.465	<0.001^*^
Calcium-phosphate product	0.593	7.253	<0.001^*^
Albumin	0.073	0.702	0.484
KT/V	−0.395	3.542	<0.001^*^
iPTH	0.334	3.396	<0.001^*^
25(OH)D	0.232	2.435	0.342

*LDL, low-density lipoprotein; HDL, high-density lipoprotein; KT/V, urea clear index; iPTH, intact parathyroid hormone; 25-(OH)D, 25-Hydroxy vitamin D*.

* *p <0.05*.

**Table 4:** Multivariate regression analysis of regulatory factors of FGF23.

**Variables**	**β**	**SE**	***P***
Calcium	0.354	0.641	0.001^*^
Phosphate	0.429	0.285	<0.001^*^
iPTH	0.235	0.310	0.353
KT/V	−0.218	0.232	0.053
Calcium-phosphate product	0.600	0.104	<0.001^*^

*Take the variables which satisfied P <0.3 in the univariate correlation analysis as candidates, multivariate regression analysis has been done. Then we adopt stepwise regression, taking P <0.05 as inclusion criteria and P > 0.10 as exclusion criteria. When calcium-phosphate product was taken into consideration, calcium and phosphate is excluded. The other results are derived from the global entry method*.

* *p <0.05*.

### Echocardiographic Parameters, BP, and Ultrafiltration

**Figure 2:**
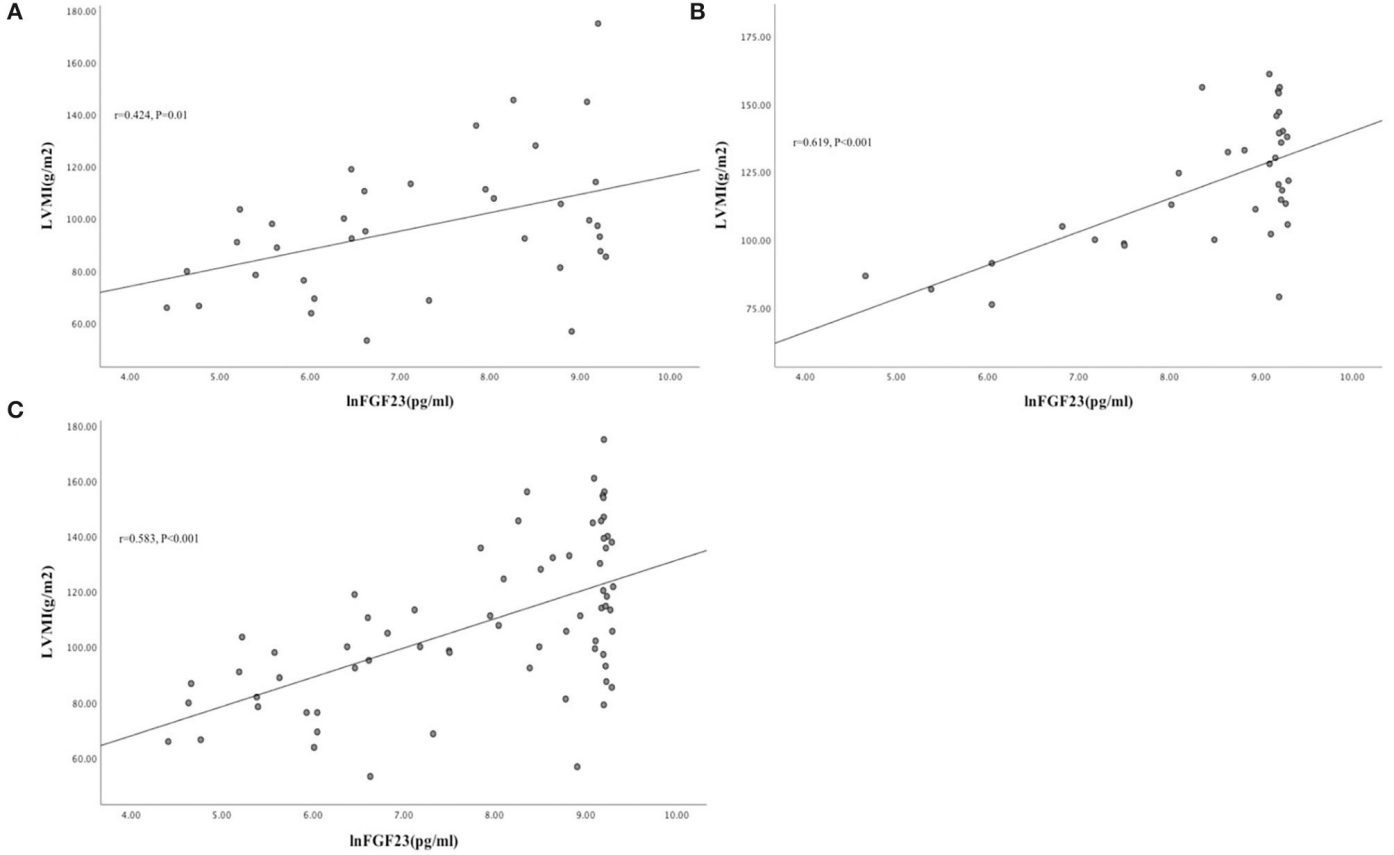
Univariate linear correlation between lnFGF23 and LVMI in INHD group **(A)**, CHD group **(B)**, and within overall patients **(C)** (Figure 3).

Patients in the INHD group displayed improvement in the cardiac structure and function manifested by lower LVMI (98.7 ± 32.1 vs. 113.9 ± 69.2 g/m^2^) (*P* < 0.01), LAD, LVDD, and LVPW, slightly higher LVEF (63.75 ± 3.3 vs. 52.44 ± 5.29%) with a non-significantly different *E*/*A* ratio (0.78 ± 0.28 vs. 0.67 ± 0.13) (*P* = 0.058) compared with the CHD group (Table 5). Univariate linear regression showed lnFGF23 was significantly correlated with LV mass index (LVMI) in the INHD group (*r* = 0.424, *P* = 0.01), CHD group (*r* = 0.619, *P* < 0.001), and within overall patients (*r* = 0.583, *P* < 0.001) (Figure 2).

**Table 5:** Comparison of echocardiographic parameters between CHD and INHD.

**Item**	**INHD (*n* = 40)**	**CHD (*n* = 40)**
LVMI (g/m^2^)	98.7 ± 32.1^*^	113.9 ± 69.2
LAD (mm)	33.25 ± 5.57^*^	41.44 ± 8.40
LVDD (mm)	45.63 ± 4.37^*^	53.33 ± 6.78
LVPW (mm)	9.84 ± 1.91^*^	11.53 ± 1.65
LVEF (%)	63.75 ± 3.3	52.44 ± 5.29
E/A	0.78 ± 0.28	0.67 ± 0.13

*LVMI, left ventricular mass index; LAD, left atrial dimension; LVDD, left ventricular end diastolic dimension; LVPW, left ventricular posterior wall; LVEF, left ventricular ejection fraction*.

* *p <0.05*.

**Figure 3:**
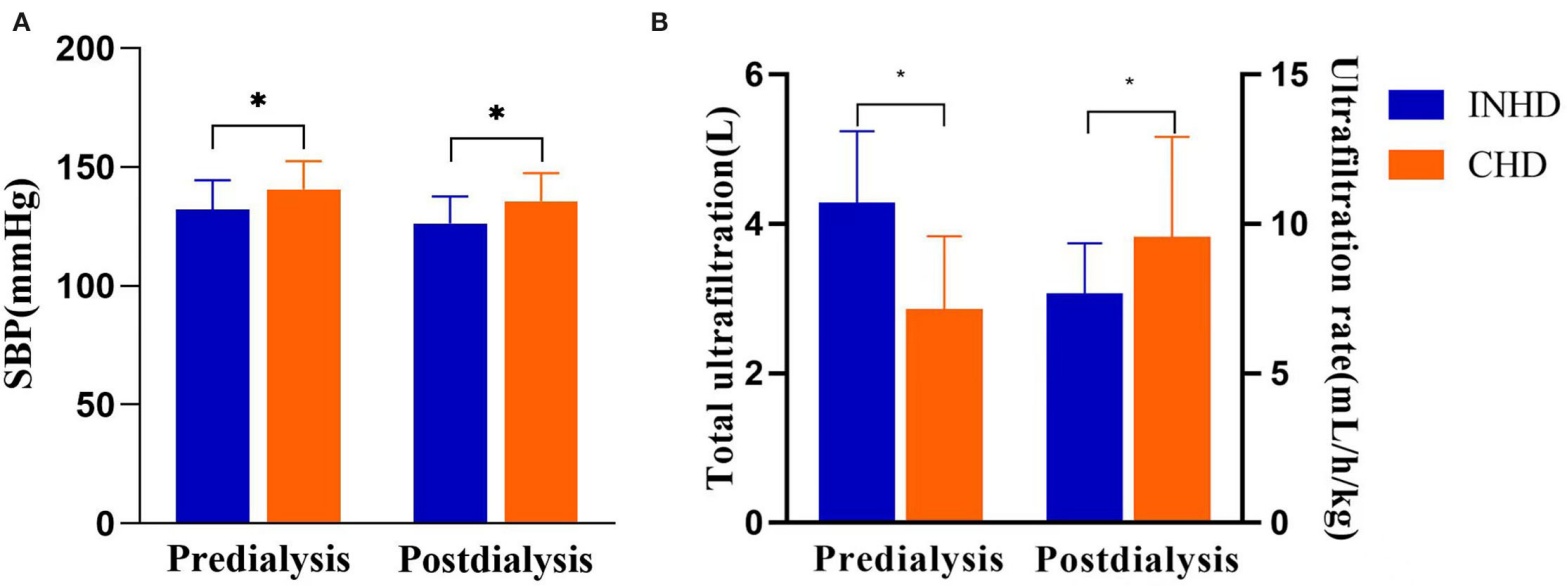
**(A)** Comparison of systolic blood pressure between patients undergoing INHD and CHD. **(B)** Comparison of volume control between patients undergoing INHD and CHD. *P < 0.05.

The INHD group had better BP control, with lower pre-dialysis SBP (132 ± 12 vs. 140 ± 11 mmHg) (*P* = 0.028) and post-dialysis SBP (126 ± 11 vs. 136 ± 13 mmHg) (*P* = 0.032). INHD group also had higher total ultrafiltration volume (4.2 ± 1.0 vs. 2.8 ± 1.2 L) (*P* = 0.003) and slower ultrafiltration rate (7.5 ± 1.8 vs. 9.3 ± 3.5 ml/h/kg) (*P* = 0.029; Figure 3).

### Survival Analysis

**Figure 4:**
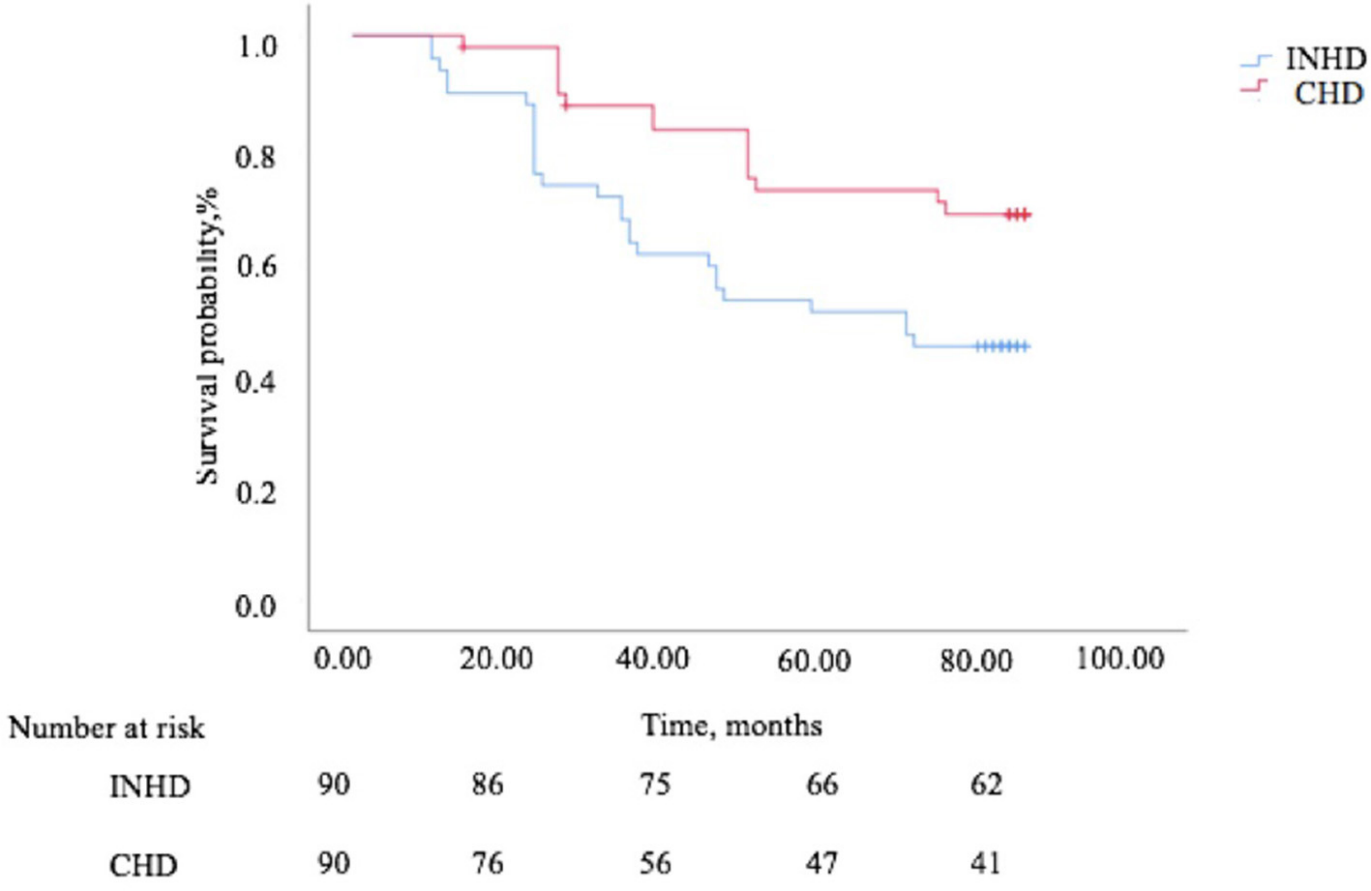
The Kaplan–Meier survival curves comparing patients undergoing INHD and CHD in terms of mortality.

The overall survival rate of INHD was better than CHD as depicted using the Kaplan–Meier method (χ^2^ = 6.860, *P* = 0.009) (Figure 4). Multivariable Cox proportional analyses were adjusted for age, gender, primary disease, ferritin, etc. Serum phosphate, dialysis vintage, and albumin were independent predictors of all-cause mortality in patients with ESRD. The hemodialysis modality of INHD was a protective factor for survival. However, iFGF23 and KT/V were not significantly associated with mortality in this adjusted model (Table 6). Compared with patients in the first FGF23 quartile, there was no trend toward an association between elevated iFGF23 and mortality in the adjusted model shown in Table 7.

**Table 6:** Cox regression analyses of factors predicting all-cause mortality.

**Variables**	**β**	**SE**	***P***	**HR**	**95%CI**
Age	0.092	0.463	0.842	1.097	0.442, 2.719
Dialysis vintage	0.079	0.040	0.047^*^	1.082	1.001, 1.170
Hemoglobin	−0.265	0.390	0.496	0.767	0.357, 1.647
Albumin	−1.068	0.495	0.031^*^	0.344	0.130, 0.906
Calcium	1.037	0.811	0.201	2.821	0.575, 13.838
Phosphate	0.545	0.366	0.001^*^	2.304	1.842, 3.532
iPTH	0.011	0.325	0.937	1.011	0.535, 1.911
KT/V	−1.552	0.452	0.230	0.932	0.087, 1.514
LnFGF23	0.418	0.562	0.457	1.519	0.505, 4.567
INHD	−0.814	0.323	0.012^*^	0.443	0.235, 0.834

*Take the variables which satisfied P <0.3 in the univariate cox regressive and some other factors as adjustment factors, a multivariable Cox proportional hazards regression has been done. Multivariable Cox regression analyses were adjusted for age, gender, dialysis vintage, hemoglobin, albumin, calcium, phosphate, KT/V, lnFGF23, ferritin. iPTH, intact parathyroid hormone; KT/V, urea clear index; FGF23, Fibroblast growth factor 23; INHD, In-center nocturnal hemodialysis*.

* *p <0.05*.

**Table 7:** Adjusted HR (95% CI) for mortality by quartile of FGF23.

	**Quartile of FGF23**			
**Model**	** <2,235 pg/ml**	**2,235–4,791 pg/ml**	**4,791–8,503 pg/ml**	**>8,503 pg/ml**
No adjustment	1 (ref)	1.967 (1.324, 2.500)	1.073 (0.519, 2.220)	1.168 (0.544, 2.508)
Model 1	1 (ref)	1.153 (0.972, 1.365)	0.841 (0.278, 2.546)	1.087 (0.362, 3.267)
Model 2	1 (ref)	1.139 (0.992, 1.327)	0.943 (0.852, 1.034)	0.913 (0.765, 1.042)

*Model 1 was adjusted for age, gender, prior cardiovascular disease, dialysis vintage, hemoglobin, albumin, calcium, phosphate, and ferritin*.

*Model 2 was adjusted for Model 1 plus dialysis modality*.

## Discussion

Nocturnal hemodialysis was first reported by Shaldon in 1963. Thrice-weekly in-center nocturnal hemodialysis has been adopted as the standard therapeutic strategy in Tassin since 1968, and more than 50 years of long-term experience has been gained until now (14). Our center initiated the INHD program in February 2009. Consistent with the previous studies, patients in the INHD group showed increased urea clearance (KT/V), lower serum phosphorus, and significantly reduced requirements of EPO and phosphate binders, together with the improvement of echocardiographic parameters and mortality (11). In addition, for the first time, we found that circulating iFGF23 was significantly reduced in INHD compared to CHD, which might work as a bridge to connect the CKD–MBD with left ventricular hypertrophy and all-cause mortality.

First, the potential regulatory factors of iFGF23 in dialysis patients were evaluated. Although there were many studies focused on the regulation of elevated FGF23 in chronic renal failure, the conclusions were inconsistent. It is well known that phosphate load could enhance FGF23 secretion (15), but the molecular mechanism of how the osteocytes and osteoblasts sensed the extracellular phosphorus and stimulate the downstream FGF23 expression remains unclear. 1,25(OH)_2_D_3_ was a positive regulator on FGF23. It can stimulate the transcription of FGF23 through binding to a vitamin D-responsive element on the FGF23 promoter (16). To avoid the interference of medication on circulating iFGF23 levels in the two groups of patients, we collected the medication data and found that there was no significant difference in the patient numbers taking active vitamin D and the dosage of these categories of drugs between the two groups, but the dosage of calcium-containing phosphate binders was significantly reduced in the INHD group. Therefore, the decreased level of iFGF23 in INHD was not related to the difference in active vitamin D. PTH is another potential regulator of FGF23 in chronic renal failure since PTH and FGF23 were both elevated at the same time. Lavi-Moshayoff et al. suggested that PTH could stimulate the FGF23 expression *in vitro*, but other studies could not prove the direct regulation of FGF23 (17). Our previous study found that significantly elevated PTH could not stimulate the expression of FGF23 in 1α-hydroxylase knockout mice with 1,25(OH)_2_D_3_ deficiency and hypocalcemia (18). Similarly, the circulating iFGF23 in double knockout CKD mice with impaired production of PTH and calcitriol was significantly abated and could be restored by administration of a high calcium high phosphorus diet, suggesting that neither PTH nor active vitamin D was indispensable for elevated iFGF23 in chronic renal failure (18, 19).

Multivariate analysis showed that only phosphorus, calcium, and calcium-phosphorus products were positively correlated with FGF23. Among them, it was an important finding that the serum calcium was positively correlated with FGF23 under the circumstances that the serum total calcium was not statistically different between the two groups, which indicated that calcium could positively regulate FGF23 by augmenting calcium load and calcium-phosphorus product before the serum calcium rose beyond the normal range. In our previous experimental study (18), we found that circulating iFGF23 significantly increased 6 h after calcium chloride administration in wild type and PTH-deficient mice. *In vitro*, we found that after adding different concentrations of calcium (1, 2, 4, 6, and 8 mmol/L) to the MC3T3-E1 osteoblast cell line, FGF23 transcription assayed by reporter gene reached maximum activation at 6 mmol/L calcium, and this effect could be inhibited by calcium channel blocker, which showed that extracellular calcium could directly regulate the transcription of FGF23. In a previous *in vivo* study, it was found that at least 50 mg/L of serum phosphorus was required for calcium to promote FGF23 secretion, and at least 80 mg/L of calcium was required for phosphorus to stimulate FGF23 expression (20). Therefore, in dialysis patients, the abnormally elevated calcium-phosphorus product rather than a single mineral ion might be the most essential regulatory factor to enhance the serum iFGF23 secretion. With regard to the underlying molecular mechanism, we speculate that the osteocytes could sense the calcium and phosphorus overload, and extracellular calcium and phosphorus deposited in the skeleton microenvironment might stimulate the secretion of circulating iFGF23 through a probably sodium/phosphate cotransporter (PiT1,2) or FGF receptor (FGFR1) dependent mechanism (21, 22).

It is not a novel idea to emphasize the calcium-phosphorus product in CKD–MBD. However, we suggest this finding has important clinical implications since there are many available strategies to modify the calcium-phosphorus product, which is an independent determining factor of serum iFGF23 levels in dialysis patients. At present, several clinical studies have supported the assumption that lowering calcium-phosphorus products led to an abated serum iFGF23 level in patients with CKD. For example, non-calcium-containing phosphate binder sevelamer was proved to significantly reduce serum iFGF23 compared with calcium-containing phosphate binder, through the mechanism of reducing elemental calcium intake and calcium-phosphorus product (23). Cinacalcet can also attenuate calcium-phosphate products and decrease serum iFGF23 levels in patients with CKD (24).

Two elegant experimental studies have proved that FGF23 can directly induce left ventricular hypertrophy *in vivo* and *in vitro*. The non-classic effect of FGF23 on the heart was independent of Klotho but through binding to FGFR4 on cardiomyocytes. FGF23 activated the PLCγ phosphorylation and the calcineurin/NFAT signaling pathway, finally upregulating the expression of cardiac hypertrophy-related genes (25, 26). There were also different views on whether FGF23 could directly cause myocardial damage (27, 28). Takashi et al. found that although serum iFGF23 was elevated in animal models and patients with X-linked hereditary hypophosphatemia, notable cardiac hypertrophy was not found (27). A recent meta-analysis compared the association between cardiovascular events and FGF23 in the general population, non-dialysis patients with CKD, and dialysis patients, although serum FGF23 went up with the progression of CKD, there was no powerful evidence that increased FGF23 contribution to heart failure, stroke, or myocardial infarction either in the overall or any of the three separate groups (29). To interpret the paradoxical phenomena, we surmised that there were many factors leading to cardiac hypertrophy in uremic patients, while elevated FGF23 was only one of the complex risk factors. Consistent with our previous study and the findings of Culleton et al., this cohort study showed that INHD could significantly improve echocardiographic parameters and reduce left ventricular hypertrophy (12, 13, 30). Based on the positive correlation between serum iFGF23 and LVMI in this study, we speculated that INHD might improve left ventricular hypertrophy partially by reducing serum iFGF23 levels. Better blood pressure control, slower ultrafiltration rate, and more optimized volume management might also be involved.

Many studies had shown that the increased FGF23 in patients with CKD was closely related to the worsened mortality (4, 31). The underlying mechanism comprises not only FGF23 induced myocardial toxicity but also aggravating inflammation, immunologic dysfunction, anemia, etc. (32, 33). Therefore, lowering circulating FGF23 holds the promise to bring survival benefits. Although in some clinical trials, the hypothesis seemed to be true, the causal relationship was hard to confirm. If we take overall hemodialysis patients into consideration, the conclusion becomes ambiguous. J-DOPPS found that as the dialysis vintage increased, the association between iFGF23 and mortality weakened and disappeared in the highest tertile (>9.4 years), indicating that the predictive value of iFGF23 as a prognosis biomarker was blunted with long-term dialysis (34). In a Dutch FGF23 cohort from the CONTRAST study, no association between a single value of cFGF23 and all-cause mortality was found. In addition, decreased cFGF23 in the hemodiafiltration group was not associated with improved survival compared to the hemodialysis group (35). In this study, the association between attenuated iFGF23 and reduced mortality also could not be established in patients with INHD. A plausible explanation was that most of the patients we enrolled had a longer dialysis vintage (average 8.3 years), and serum iFGF23 was already at a very high level, which probably masked the survival benefit.

Consistent with previous studies, this study showed that serum phosphorus, dialysis vintage, and albumin were independent predictors of mortality in long-term hemodialysis patients. In addition, no correlation between all-cause mortality and hemoglobin, KT/V was found, which suggested that the improved survival of INHD was not due to the ameliorative dialysis adequacy of small-molecule uremic solutes. We believe that INHD persistently conveyed a protective effect on survival through modifying the CKD–MBD parameters, such as hyperphosphatemia, representing an FGF23 independent mechanism involved.

This study has the following limitations: First, this study is a single-center retrospective cohort study with a small number of patients. Some relevant parameters were missing or incomplete, such as c-terminal FGF23 (cFGF23), BNP, pro-BNP, echocardiographic data, and cardiovascular mortality. Second, the complex internal environment of hemodialysis patients and multiple regulatory mechanisms made the causal relationship between those variables could not be explicitly judged. Third, the majority of clinical data were collected and analyzed during the enrollment period as well as at the 1-year follow-up. Further studies on longitudinal changes in those parameters might greatly enhance the scientific value and make the conclusion robust. Fourth, most of the patients we enrolled had long dialysis vintage with no residual kidney function, although there was no statistically significant difference between the two groups, the outcomes still might be biased. Further studies on incident hemodialysis patients with shorter dialysis vintages might yield some different results.

In summary, the INHD group presented with modification of CKD–MBD parameters, including serum phosphorus, calcium-phosphorus product, and iFGF23 compared with the CHD in dialysis patients. The calcium-phosphorus product is the most important and independent regulatory factor of iFGF23 in hemodialysis patients. Decreased serum iFGF23 in patients with INHD might be partially involved in attenuating cardiac structural parameters. INHD significantly reduces all-cause mortality, which was not correlated with abated circulating iFGF23 suggesting other FGF23 independent mechanisms were involved and remained to be clarified.

## Data Availability Statement

The original contributions presented in the study are included in the article/Supplementary Material, further inquiries can be directed to the corresponding authors.

## Ethics Statement

The studies involving human participants were reviewed and approved by Ethics Committee of Changzheng Hospital. The patients/participants provided their written informed consent to participate in this study.

## Author Contributions

MK, JC, CX, and BD conceived and designed the protocol and study. MK, JC, and LL identified cases for eligibility. MK, XT, ZM, and JL extracted data of included cases. MK and LF performed the data analysis with assistance of CM, YL, and BD. MK and BD drafted the article for important content. BD and YL reviewed and revised this manuscript. All authors read and approved the final manuscript.

## Funding

This work was supported by the National Natural Science Foundation of China under Grant No. 81970640.

## Conflict of Interest

The authors declare that the research was conducted in the absence of any commercial or financial relationships that could be construed as a potential conflict of interest.

## Publisher's Note

All claims expressed in this article are solely those of the authors and do not necessarily represent those of their affiliated organizations, or those of the publisher, the editors and the reviewers. Any product that may be evaluated in this article, or claim that may be made by its manufacturer, is not guaranteed or endorsed by the publisher.

## Supplementary Material

Supplementary Figure S1 Study flow diagram.

Supplementary Table S1 Baseline biochemical parameters of patients undergoing hemodialysis.
