# Supplementary figures and images for: In-center Nocturnal Hemodialysis Reduced the Circulating FGF23, Left Ventricular Hypertrophy, and All-Cause Mortality: A Retrospective Cohort Study
Source: Front Med (Lausanne). 2022 Jun 21;9:912764. doi: 10.3389/fmed.2022.912764 (PMC9253468; doi:10.3389/fmed.2022.912764)

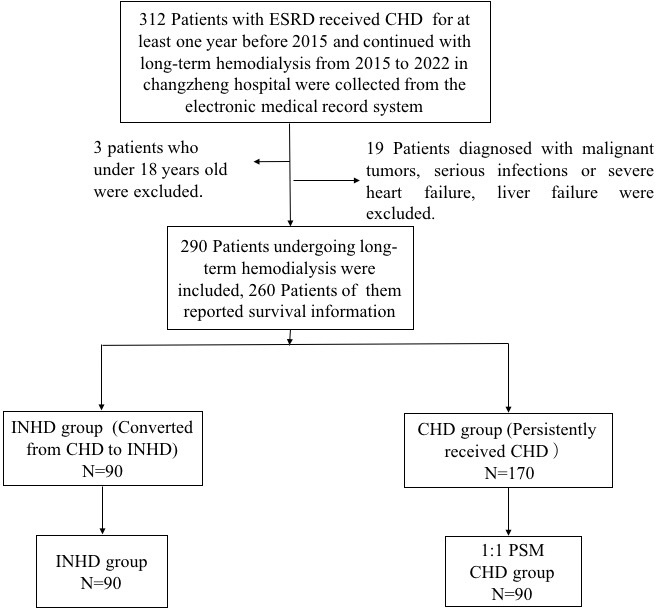

Supplement: Supplementary Figure S1 — Study flow diagram. [file Image_1.JPEG]
