# Supplementary material for: In-center Nocturnal Hemodialysis Reduced the Circulating FGF23, Left Ventricular Hypertrophy, and All-Cause Mortality: A Retrospective Cohort Study
Source: Front Med (Lausanne). 2022 Jun 21;9:912764. doi: 10.3389/fmed.2022.912764 (PMC9253468; doi:10.3389/fmed.2022.912764)
Supplement: Supplementary Table S1 — Baseline biochemical parameters of patients undergoing hemodialysis. [file Table_1.DOCX]

**TABLE S1.** Baseline biochemical parameters of patients undergoing hemodialysis

| **Item** | **INHD(n=90)** | **CHD(n=90)** |  |
| --- | --- | --- | --- |
| iFGF23  iPTH  Cholesterol(mmol ∙ L^-1^)  Triglycerides(mmol ∙ L^-1^)  LDL cholesterol (mmol ∙ L^-1^)  HDL cholesterol (mmol ∙ L^-1^)  Phosphate(mmol ∙ L^-1^)  Calcium (mmol ∙ L^-1^)  Calcium-phosphate product  (mmol^2^ ∙ L^-2^)  Albumin(g ∙ L^-1^)  KT/V  25(OH)D(ng ∙ ml^-1^)  Hemoglobin(g ∙ L^-1^)  Ferritin(ug ∙ L^-1^)  β2-MG(mg ∙ L^-1^) | 8723.58(3578.39-10958.52)  482.92±213.20  3.61±0.72  2.34±1.56  1.92±0.63  0.86±0.24  1.98.±0.69  2.41±0.29  5.12±1.47  42.0±4.13  1.53±0.42  27.38±9.13  112.57±14.36  210.25±143.01  17.3±4.2 | 8238.12(3927.12-10723.79)  453.41±198.53  3.65±0.83  2.28±1.52  2.03±0.67  0.90±0.25  2.02±0.64  2.48±0.36  5.09±1.53  41.27±4.25  1.45±0.38  26.41±9.22  111.63±14.09  223.51±123.01  16.9±4.9 |  |

*IFGF23,intact Fibroblast growth factor 23；IPTH, intact parathyroid hormone; LDL, low-density lipoprotein; HDL, high-density lipoprotein;* *KT/V, urea clear index; 25-(OH)D, 25-Hydroxy vitamin D ; β2-MG, β2-Microglobulin . *P<0.05.*
